# Profiling of Parkin-Binding Partners Using Tandem Affinity Purification

**DOI:** 10.1371/journal.pone.0078648

**Published:** 2013-11-11

**Authors:** Alessandra Zanon, Aleksandar Rakovic, Hagen Blankenburg, Nadezhda T. Doncheva, Christine Schwienbacher, Alice Serafin, Adrian Alexa, Christian X. Weichenberger, Mario Albrecht, Christine Klein, Andrew A. Hicks, Peter P. Pramstaller, Francisco S. Domingues, Irene Pichler

**Affiliations:** 1 Center for Biomedicine, European Academy Bozen/Bolzano (EURAC), Bolzano, Italy, Affiliated Institute of the University of Lübeck, Lübeck, Germany; 2 Institute of Neurogenetics, University of Lübeck, Lübeck, Germany; 3 Max Planck Institute for Informatics, Saarbrücken, Germany; 4 Department of Bioinformatics, Institute of Biometrics and Medical Informatics, University Medicine Greifswald, Greifswald, Germany; 5 Department of Neurology, General Central Hospital, Bolzano, Italy; 6 Department of Neurology, University of Lübeck, Lübeck, Germany; Uppsala University, Sweden

## Abstract

Parkinson's disease (PD) is a progressive neurodegenerative disorder affecting approximately 1–2% of the general population over age 60. It is characterized by a rather selective loss of dopaminergic neurons in the substantia nigra and the presence of α-synuclein-enriched Lewy body inclusions. Mutations in the *Parkin* gene (*PARK2*) are the major cause of autosomal recessive early-onset parkinsonism. The Parkin protein is an E3 ubiquitin ligase with various cellular functions, including the induction of mitophagy upon mitochondrial depolarizaton, but the full repertoire of Parkin-binding proteins remains poorly defined. Here we employed tandem affinity purification interaction screens with subsequent mass spectrometry to profile binding partners of Parkin. Using this approach for two different cell types (HEK293T and SH-SY5Y neuronal cells), we identified a total of 203 candidate Parkin-binding proteins. For the candidate proteins and the proteins known to cause heritable forms of parkinsonism, protein-protein interaction data were derived from public databases, and the associated biological processes and pathways were analyzed and compared. Functional similarity between the candidates and the proteins involved in monogenic parkinsonism was investigated, and additional confirmatory evidence was obtained using published genetic interaction data from *Drosophila melanogaster*. Based on the results of the different analyses, a prioritization score was assigned to each candidate Parkin-binding protein. Two of the top ranking candidates were tested by co-immunoprecipitation, and interaction to Parkin was confirmed for one of them. New candidates for involvement in cell death processes, protein folding, the fission/fusion machinery, and the mitophagy pathway were identified, which provide a resource for further elucidating Parkin function.

## Introduction

Parkinson's disease (PD) is a progressive neurodegenerative disorder affecting approximately 1–2% of the general population over age 60 [1]. It is characterized clinically by tremor, rigidity, reduced motor activity (bradykinesia), and postural instability [2] and pathologically by loss of dopaminergic neurons in the *substantia nigra pars compacta* and the presence of α-synuclein positive inclusions in the cytoplasm of neurons, termed Lewy bodies [3], [4]. Most cases are idiopathic or late-onset PD (>85% of all cases), whereas <10% of cases are familial forms. The identification and characterization of genes that cause heritable forms of the disease have provided important insights into the pathways involved in dopaminergic neurodegeneration. Mutations in the *Parkin* gene (*PARK2*) represent the most common known cause of early-onset parkinsonism (10 to 20%) [5]. The Parkin protein is an E3 ubiquitin ligase responsible for the transfer of activated ubiquitin molecules to a protein substrate [6]. This ubiquitination process has various functional consequences in addition to the protein degradation by the 26S proteasome, including regulation of receptor trafficking, cell cycle progression, gene transcription, DNA repair, and immune responses [7].

Studies in *Drosophila melanogaster* revealed compelling evidence for a role of Parkin in the maintenance of mitochondrial function [8]. Genetic interaction between *Parkin* and *PINK1*, mutations of which also cause early-onset parkinsonism, indicated that both genes are acting in a common pathway. Loss of one of these two genes results in mitochondrial pathology and muscle and dopaminergic neuron degeneration. Overexpression of *Parkin* rescues the phenotypes caused by *PINK1* deficiency, but not vice versa, indicating that *Parkin* intervenes downstream of *PINK1* [9], [10]. In addition, genetic interactions between *Parkin* and *PINK1* and genes encoding components of the mitochondrial fission/fusion machinery indicate an involvement of the *PINK1/Parkin* pathway in the regulation of mitochondrial dynamics [11], [12].

Parkin is at steady state essentially cytosolic, and recent work has shown that it selectively and rapidly translocates from the cytosol to depolarized mitochondria with low membrane potential and subsequently induces their autophagic removal in a process called mitophagy [13]–[16].

Increasing our knowledge about the interactions between Parkin and other cytoplasmic and mitochondrial proteins will provide further biological insights into Parkin function and the intricate relationships between the multiple roles of Parkin. The identification of such Parkin-binding proteins may have a general role in the pathogenesis of PD and elucidate novel therapeutic targets.

In this study, we report a comprehensive set of novel candidate Parkin-binding proteins identified by Tandem Affinity Purification (TAP)/mass spectrometry (MS) interaction screens. Following the established “guilt by association” strategy, where proteins/genes are prioritized if they are found to be related to known disease genes and processes [17]–[19], a set of “seed” proteins known to be related to genetic parkinsonism was used to prioritize the candidate Parkin-binding proteins. In particular, this set of proteins provided the basis for the prioritization of candidate proteins based on the known interactions to these proteins. In addition, it was used in an analysis of PD-related pathways and processes and in the prioritization of the candidate Parkin-binding proteins based on their functional relationships. The candidate proteins were also compared to complementary experimental data from genetic interaction screens in *Drosophila melanogaster* and genome-wide association studies (GWAS) in humans. Our study identified novel candidate Parkin-binding proteins for involvement in cell death processes, protein folding and response to unfolded protein, the fission/fusion machinery, and the mitophagy pathway, and the combined results of the bioinformatics analyses were used to prioritize them into different selection levels.

## Results

Protein-protein interaction data for the candidate Parkin-binding proteins obtained from the TAP experiments and the proteins known to cause heritable forms of parkinsonism were derived from public databases, and the respective biological processes and pathways were analyzed and compared. Network models were applied to investigate the functional relationships between the candidate Parkin-binding proteins and the proteins related to monogenic parkinsonism. In addition, the candidate dataset was compared to results from genetic interaction screens in *Drosophila* and human GWAS. The candidate proteins were prioritized into different selection levels, which were compared to the results of an independent gene prioritization approach. Finally, two candidates were tested for interaction to Parkin by co-immunoprecipitation.

### TAP results and protein datasets

TAP-tagged Parkin containing protein complexes were purified in a two-stage purification process of protein extracts prepared from whole cell lysates and cytosolic and mitochondrial fractions from HEK293T and SH-SY5Y cells and analyzed by MS. The TAP experiments resulted in different protein datasets listed in Table 1 (ParkinTAP datasets). In total, 203 unique peptides were identified as candidate Parkin-binding proteins (Table 1; ParkinTAP candidates); approximately 50% of the candidate proteins were identified in the mitochondrial fractions (Mito dataset) and 50% in the cytosolic fractions (Cyto dataset), with an overlap of 49 proteins between the fractions.

**Table 1:** Protein datasets.

Label	Description	Size
**ParkinTAP datasets**		
WholeCellsNT-293T	HEK293T, Not Treated, Whole Cells	97
MitoNT-293T	HEK293T, Not Treated, Mitochondrial Fraction	65
CytoNT-293T	HEK293T, Not Treated, Cytosolic Fraction	55
MitoT-293T	HEK293T, Treated, Mitochondrial Fraction	18
CytoT-293T	HEK293T, Treated, Cytosolic Fraction	22
MitoT-SH-SY5Y	SH-SY5Y, Treated, Mitochondrial Fraction	54
CytoT-SH-SY5Y	SH-SY5Y, Treated, Cytosolic Fraction	53
**Combined datasets**		
ParkinTAP candidates	Union of all ParkinTAP datasets: WholeCellsNT-293T, MitoNT-293T, CytoNT-293T, MitoT-293T, CytoT-293T, MitoT-SH-SY5Y, CytoT-SH-SY5Y	203
Mito	Union of MitoNT-293T, MitoT-293T and MitoT-SH-SY5Y	99
Cyto	Union of CytoNT-293T, CytoT-293T and CytoT-SH-SY5Y	94
**External datasets**		
MonogenicPD	Proteins encoded by genes causing monogenic parkinsonism [20]	9
Pink1TAP	PINK1-interacting candidates identified by TAP [21]	17
ParkinIP	Parkin interacting proteins from HNet	77
MonogenicPDIP	Interacting partners of MonogenicPD proteins from HNet	668
PINK1IP	PINK1-interacting proteins from HNet	44
RelatedPD	Union of ParkinIP and MonogenicPD	80
**Comparison of datasets**		
ParkinTAP ∩ ParkinIP*	Overlap between ParkinTAP candidates and Parkin interactors in HNet	4(3)**
ParkinTAP ∩ MonogenicPD	Overlap between ParkinTAP candidates and MonogenicPD	1(0)
ParkinTAP ∩ MonogenicPDIP	Overlap between ParkinTAP candidates and MonogenicPDIP	40(39)
ParkinTAP ∩ Pink1TAP	Overlap between ParkinTAP and Pink1TAP candidates	15(15)
ParkinIP ∩ MonogenicPD	Overlap between Parkin interactors in HNet and in MonogenicPD	6(5)

* dataset intersection (∩).

** in brackets: set size excluding Parkin.

In addition, the following datasets were used in the analyses: MonogenicPD, which includes proteins encoded by genes implicated in monogenic forms of parkinsonism [20], Pink1TAP, which provides a list of candidate PINK1-interacting proteins identified in a previous TAP study [21], and ParkinIP, PINK1IP, and MonogenicPDIP, which include proteins known to interact with Parkin, PINK1, and proteins from MonogenicPD, respectively. The dataset RelatedPD includes the ParkinIP and MonogenicPD datasets. The previously reported Pink1TAP dataset mostly overlaps with the ParkinTAP candidates of the present study, with the exception of PINK1 itself and CDC37 (cell division cycle 37 homolog). The database identifiers of the proteins present in each dataset are provided in Table S1.

### Protein-protein interactions

The protein interactions of Parkin and MonogenicPD were investigated based on the human interactome network (HNet). We analyzed the interaction network within the RelatedPD dataset, which includes 80 proteins and 206 binary interactions, out of 307 interactions in total. Six of the nine MonogenicPD proteins are Parkin interactors in HNet (ParkinIP ∩ MonogenicPD in Table 1), and only UCHL1, FBXO7, and ATP13A2 from MonogenicPD do not interact directly with Parkin. Nevertheless, UCHL1 and FBXO7 interact with ParkinIP proteins, and therefore the RelatedPD subnetwork consists of a single connected component when ATP13A2, which is responsible for Kufor-Rakeb syndrome, a form of parkinsonism with dementia and juvenile disease onset [22], is excluded.

We investigated also the interactions between the proteins in ParkinTAP, and out of the 203 candidates, 193 are part of a single large connected component, and only 10 do not interact with the other ParkinTAP candidates (Table S2).

For each ParkinTAP candidate, the shortest path network distance (ND) to Parkin and to MonogenicPD proteins was computed in SpNet, which is a subnetwork of HNet, including the ParkinTAP candidates, ParkinIP, MonogenicPD, MonogenicPDIP and all proteins in the interconnecting shortest paths between ParkinTAP and MonogenicPD. In total, it includes 4,009 proteins and 290,496 interactions, where most of the interactions (268,484) are expanded complexes. Table 2 shows the ND to Parkin and the minimum network distance to MonogenicPD proteins for a selection of candidates, the results for all ParkinTAP candidates are available in Table S3 (ND = 1 corresponds to direct interaction, ND = 2 indicates one intermediate in the shortest path). In this network, only three ParkinTAP candidates are known Parkin-binding proteins (DNAJA1, HSPA1A, HSPA8) [23], and 164 candidate proteins interact with Parkin through one intermediate protein (Parkin ND = 2). In total, 40 candidates are MonogenicPDIP, and six of them interact with two different MonogenicPD proteins (MonogenicPD #ND = 2). Most of the interactions to MonogenicPD (28 of 40) include UCHL1, which was not confirmed since first described in 1998 [24], and 25 of these involve a large complex consisting of UCHL1 and 166 additional proteins [25]. Ten ParkinTAP candidates interact with PARK7 (DJ-1), three interact with SNCA, and one interacts with LRRK2 (Table 2; column iMonogenicPD).

**Table 2:** Summary of results for ParkinTAP candidates passing the first seven selection levels.

Rank	Entrez Gene ID	HGNC Symbol	Parkin ND	MonogenicPD ND	MonogenicPD #ND	iMonogenicPD	Not Complex	Pink1 TAP	HNet Degree	GOComp	FunSim MonogenicPD	GOSlimPD	ParkinGS	Pink1GS	CalmodulinIP	Selection Level
1	5071	PARK2	0	0	1	PARK2	–	–	76	true	true	–	true	true	–	0
2	3301	DNAJA1	1	1	2	PARK2, UCHL1	–	–	551	true	–	true	true	–	true	0
3	3303	HSPA1A	1	1	2	PARK2, SNCA	–	true	926	–	true	true	true	true	true	0
4	3312	HSPA8	1	1	2	PARK2, UCHL1	–	true	1292	true	–	true	true	true	true	0
5	5052	PRDX1	2	1	2	UCHL1, PARK7	–	–	559	–	–	true	–	–	true	1
6	801	CALM1	2	1	2	UCHL1, SNCA	–	–	780	–	–	–	true	true	true	1
7	3181	HNRNPA2B1	2	1	2	UCHL1, PARK7	–	–	848	–	–	–	–	–	true	1
8	10845	CLPX	2	1	1	PARK7	–	–	161	true	true	–	true	true	–	2
9	10951	CBX1	3	1	1	UCHL1	–	–	235	–	true	–	true	true	–	2
10	3329	HSPD1	2	1	1	PARK7	–	true	904	true	true	true	true	true	true	2
11	60	ACTB	2	1	1	UCHL1	–	–	1016	true	true	true	true	true	true	2
12	492	ATP2B3	3	3	8	0	true	–	2	–	true	true	–	–	–	3
13	9868	TOMM70A	2	2	5	0	true	–	8	true	true	true	–	–	–	3
14	490	ATP2B1	3	2	2	0	true	–	8	–	true	true	–	–	true	3
15	23581	CASP14	3	2	5	0	–	–	29	–	true	true	true	–	–	3
16	3005	H1F0	2	2	4	0	–	–	74	–	true	true	–	–	true	3
17	7818	DAP3	2	2	7	0	–	–	125	true	true	true	–	–	–	3
18	5589	PRKCSH	2	2	5	0	–	–	127	true	true	true	true	true	true	3
19	84790	TUBA1C	2	1	1	UCHL1	–	true	266	true	–	–	true	–	true	3
20	9131	AIFM1	2	2	8	0	–	–	333	–	true	true	true	true	true	3
21	10128	LRPPRC	2	1	1	PARK7	–	true	461	true	–	–	true	true	–	3
22	213	ALB	2	1	1	UCHL1	–	–	587	true	–	true	–	–	true	3
23	10383	TUBB4B	2	1	1	UCHL1	–	–	599	true	–	true	–	true	true	3
24	7431	VIM	2	1	1	SNCA	–	–	635	–	–	true	–	–	true	3
25	7277	TUBA4A	2	1	1	UCHL1	–	–	733	true	–	true	–	–	true	3
26	3326	HSP90AB1	2	1	1	LRRK2	–	true	739	true	–	true	–	–	true	3
27	3320	HSP90AA1	2	1	1	UCHL1	–	true	757	true	–	true	–	–	true	3
28	3313	HSPA9	2	1	1	UCHL1	–	true	778	true	–	true	true	true	true	3
29	4869	NPM1	2	1	1	PARK7	–	–	813	true	–	true	–	–	true	3
30	203068	TUBB	2	1	1	UCHL1	–	true	889	true	–	–	–	true	true	3
31	3309	HSPA5	2	1	1	UCHL1	–	true	921	true	–	true	–	–	true	3
32	284110	GSDMA	–	–	–	0	–	–	–	–	true	true	–	–	–	3
33	9939	RBM8A	2	1	1	UCHL1	–	–	245	true	–	–	true	true	–	4
34	813	CALU	2	1	1	UCHL1	–	–	266	true	–	–	true	true	true	4
35	5955	RCN2	2	1	1	UCHL1	–	–	297	–	–	–	true	true	true	4
36	10240	MRPS31	2	1	1	PARK7	–	–	365	–	–	–	true	true	–	4
37	2597	GAPDH	2	1	1	UCHL1	–	–	595	–	–	–	true	true	true	4
38	5250	SLC25A3	2	1	1	UCHL1	–	–	597	–	–	–	true	true	true	4
39	498	ATP5A1	2	1	1	UCHL1	–	–	611	true	–	–	true	–	true	4
40	3032	HADHB	2	1	1	UCHL1	–	–	675	–	–	–	–	true	true	4
41	1915	EEF1A1	2	1	1	UCHL1	–	–	1079	–	–	–	true	true	true	4
42	4747	NEFL	2	1	1	UCHL1	–	–	37	true	–	–	–	–	–	5
43	84617	TUBB6	2	1	1	UCHL1	–	–	174	true	–	–	–	–	true	5
44	10165	SLC25A13	2	1	1	PARK7	–	–	648	true	–	–	–	–	true	5
45	221613	HIST1H2AA	3	2	1	0	true	–	8	–	true	–	–	–	–	6
46	539	ATP5O	2	2	6	0	–	–	63	true	true	–	true	true	–	6
47	51081	MRPS7	2	2	6	0	–	–	128	–	true	–	true	–	–	6
48	6418	SET	2	2	7	0	–	–	169	true	true	–	true	true	–	6
49	4976	OPA1	2	2	6	0	–	–	177	true	true	–	true	true	true	6
50	4741	NEFM	2	1	1	UCHL1	–	–	233	–	–	–	–	–	true	6
51	11335	CBX3	2	2	5	0	–	–	256	–	true	–	true	true	–	6
52	1975	EIF4B	2	2	8	0	–	–	300	–	true	–	–	–	–	6
53	9532	BAG2	2	2	8	0	–	–	351	true	true	–	true	true	true	6
54	9092	SART1	2	1	1	PARK7	–	–	376	–	–	–	–	–	–	6
55	9551	ATP5J2	2	2	6	0	–	–	379	true	true	–	–	–	true	6
56	10627	MYL12A	2	1	1	PARK7	–	–	385	–	–	–	–	–	true	6
57	509	ATP5C1	2	2	7	0	–	–	389	true	true	–	–	–	–	6
58	6224	RPS20	2	2	7	0	–	–	390	–	true	–	true	–	–	6
59	6137	RPL13	2	2	8	0	–	–	436	–	true	–	–	–	true	6
60	6128	RPL6	2	2	7	0	–	–	468	–	true	–	–	–	true	6
61	7203	CCT3	2	2	8	0	–	–	598	true	true	–	true	true	true	6
62	302	ANXA2	2	1	1	UCHL1	–	–	811	–	–	–	–	–	true	6
63	3185	HNRNPF	2	1	1	UCHL1	–	–	928	–	–	–	–	–	true	6
64	8363	HIST1H4J	–	–	–	0	–	–	–	–	true	–	–	–	–	6
65	7171	TPM4	3	2	3	0	–	–	12	–	–	true	–	–	–	7
66	4724	NDUFS4	3	2	3	0	–	–	44	true	–	true	–	–	–	7
67	9118	INA	2	2	6	0	–	–	82	–	–	true	–	–	–	7
68	3306	HSPA2	2	2	7	0	–	true	95	true	–	true	true	–	–	7
69	1153	CIRBP	2	2	4	0	–	–	149	–	–	true	–	true	–	7
70	8531	CSDA	2	2	7	0	–	–	167	–	–	true	true	true	–	7
71	4001	LMNB1	2	2	7	0	–	–	206	–	–	true	true	true	–	7
72	291	SLC25A4	2	2	6	0	–	–	227	true	–	true	–	–	–	7
73	811	CALR	2	2	6	0	–	–	235	true	–	true	–	–	true	7
74	6182	MRPL12	2	2	5	0	–	–	263	true	–	true	–	–	true	7
75	27339	PRPF19	2	2	7	0	–	–	275	–	–	true	true	–	–	7
76	6421	SFPQ	2	2	7	0	–	–	307	–	–	true	true	true	–	7
77	7184	HSP90B1	2	2	7	0	–	–	316	true	–	true	–	–	true	7
78	5984	RFC4	2	2	6	0	–	–	324	–	–	true	–	–	true	7
79	2521	FUS	2	2	6	0	–	–	354	–	–	true	true	true	–	7
80	4720	NDUFS2	2	2	8	0	–	–	410	–	–	true	true	true	true	7
81	6742	SSBP1	2	2	7	0	–	–	531	true	–	true	true	true	true	7
82	10642	IGF2BP1	2	2	8	0	–	–	647	–	–	true	–	–	–	7
83	3305	HSPA1L	2	2	8	0	–	true	661	true	–	true	true	true	true	7
84	708	C1QBP	2	2	7	0	–	–	661	–	–	true	true	–	true	7
85	2547	XRCC6	2	2	8	0	–	–	813	–	–	true	true	true	–	7
86	7531	YWHAE	2	2	8	0	–	–	833	true	–	true	–	–	true	7
87	8607	RUVBL1	2	2	8	0	–	–	858	–	–	true	true	–	–	7
88	4000	LMNA	2	2	8	0	–	–	871	true	–	true	true	true	true	7
89	10856	RUVBL2	2	2	8	0	–	–	960	true	–	true	true	–	–	7
90	7425	VGF	–	–	–	0	–	–	–	true	–	true	–	–	–	7

Parkin ND: Shortest path network distance to Parkin in SpNet protein-protein interaction network.

MonogenicPD ND: Minimum shortest path network distance to MonogenicPD in SpNet.

MonogenicPD #ND: Number of shortest paths to MonogenicPD with minimum value in SpNet.

iMonogenicPD: Gene symbols of interacting MonogenicPD.

Not Complex: No complex interaction with other ParkinTAP candidates.

Pink1TAP: Pink1TAP candidate.

HNet Degree: Number of protein interactions in iRefIndex.

GOComp: Logical OR of “true” values of six GOComparisons listed in Table S3.

FunSim MonogenicPD: Functional similarity ≥0.7 to a MonogenicPD protein.

GOSlimPD: Annotated to GOSlimPD or children term.

ParkinGS: Overlap with Parkin fly genetic screen.

Pink1GS: Overlap with PINK1 fly genetic screen.

CalmodulinIP: Interaction with calmodulin.

**Figure 1:**
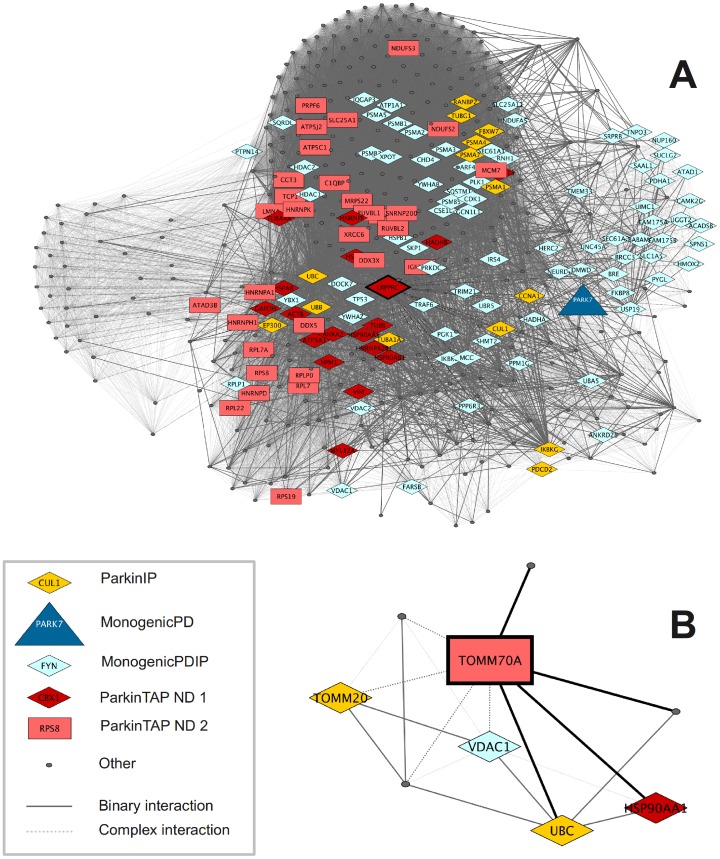
Direct protein interactions of two ParkinTAP candidates selected as exemplary proteins: LRPPRC (A) and TOMM70A (B). Proteins are represented as nodes and interactions as edges; the edges are drawn as solid and dashed lines for binary and complex interactions, respectively. Binary interactions to the selected candidates are represented by thicker edges. ParkinTAP ND X are ParkinTAP candidates at network distance X of MonogenicPD, where ParkinTAP ND 1 are direct MonogenicPD interactors. A: LRPPRC. There are many interactors of LRPPRC in iRefIndex, resulting in a dense network of complex interactions. LRPPRC interacts with MonogenicPD PARK7, as well as with 48 other ParkinTAP candidates, and the network includes 14 ParkinIP and 77 MonogenicPDIP. B: TOMM70A. Only eight proteins interact directly with TOMM70A, including one ParkinTAP candidate (HSP90AA1), two ParkinIP (TOMM20, UBC) and one MonogenicPDIP (VDAC1).

The network of the interacting partners of the ParkinTAP candidate protein LRPPRC is visualized in Figure 1A as an example for a candidate protein with many interactions. LRPPRC forms a complex with PARK7 (DJ-1), and the network is relatively dense with multiple complex interactions. It has also been identified as a candidate PINK1 interactor [21].

Another example is provided by the network of TOMM70A, which is characterized by only few interactions (Figure 1B). It consists of only eight nodes, including one ParkinTAP candidate as well as one MonogenicPDIP and two ParkinIP proteins. Interaction networks for additional candidate proteins (CLPX, PRKCSH, DAP3 and CALU) are provided in Figures S1A-S1D.

Calmodulin (CALM1) is one of the candidate Parkin-binding proteins, which interacts with two other MonogenicPD proteins (UCHL1, SNCA) in HNet. However, CALM1 is a possible artifact, since Parkin was tagged with a calmodulin binding peptide according to the TAP protocol. Therefore, any ParkinTAP candidate that is a calmodulin interactor (96 from HNet) may also be a possible TAP artifact (Table 2 and Table S3; column CalmodulinIP).

A total of 96 ParkinTAP candidates showed a significant DAPPLE score (P<0.01) indicating a high connectivity between the ParkinTAP and MonogenicPD datasets (Table S3).

### Analysis of pathways and GO biological processes related to PD

In order to identify the pathways and processes known to be involved in the pathophysiology of PD and in particular *Parkin*-linked parkinsonism, we performed enrichment analyses for the RelatedPD dataset, consisting of ParkinIP and MonogenicPD. As expected, the most significant pathways according to ConsensusPathDB were “Parkinson's disease” (P = 1.6×10^−14^), “Alpha-synuclein signaling” (P = 1.1×10^−08^), and “Role of parkin in ubiquitin-proteasomal pathway” (P = 1.5×10^−07^) (Table S4).

The most significant GO (gene ontology) biological processes were related to Parkin function in the ubiquitin-proteasome system: “protein modification by small protein conjugation” (LEA P = 2.4x10^−19^) and “protein ubiquitination” (LEA P = 1.9×10^−17^). Additional significant processes were related to apoptosis and to mitochondrial and neuronal processes: “cell death” (LEA P = 2.4×10^−14^), “regulation of cell death” (LEA P = 1.2×10^−10^), “apoptosis” (LEA P = 6.6×10^−10^), “mitochondrion organization” (LEA P = 1.4×10^−9^), “synaptic transmission” (LEA P = 2.8×10^−8^), “neuron death” (LEA P = 7.7×10^−8^), and “dopamine transport” (LEA P = 7.8×10^−8^) (Table S5).

### Pathway analysis for ParkinTAP candidates

The most significantly enriched pathways in the dataset of the 203 ParkinTAP candidate proteins were related to gene expression, in particular to RNA processing/splicing and translation: “Ribosome” (P = 2.3×10^−15^) and “Processing of Capped Intron-Containing Pre-mRNA” (P = 6.0×10^−15^). Other enriched pathways relate to protein folding, like “Prefoldin mediated transfer of substrate to CCT/TriC” (P = 2.0×10^−10^) and “Protein folding” (P = 1.9×10^−7^), or to protein processing: “Protein processing in endoplasmic reticulum” (P = 1.6×10^−8^). Pathway enrichment analysis was also performed for the Mito and Cyto datasets separately. In the Mito dataset, the most significant pathways were related to protein folding and oxidative phosphorylation, whereas in the Cyto dataset the most enriched pathways were related to gene expression. Detailed pathway enrichment results, including the ParkinTAP candidate proteins for each enriched pathway, are provided in Table S6.

### Analysis of GO biological processes for ParkinTAP candidates

Of the 203 ParkinTAP candidate proteins, 175 were annotated with a GO biological process. The most well represented biological process categories were related to RNA processing and translation, like “translational elongation” (LEA P = 1.3×10^−21^) and “nuclear mRNA splicing, via spliceosome” (LEA P = 1.1×10^−19^). Several processes related to protein folding and complex assembly were also significantly enriched, like “protein folding” (LEA P = 7.9×10^−17^), “response to unfolded protein” (LEA P = 1.2×10^−7^), or “cellular protein complex assembly” (LEA P = 8.8×10^−6^), as well as mitochondrial processes like “mitochondrial transport” (LEA P = 8.2×10^−6^). The Mito dataset contained significant terms related to mitochondrial function, which were specific to this dataset: “electron transport chain” (LEA P = 7.2×10^−9^) and “oxidative phosphorylation” (LEA P = 2.7×10^−5^). Detailed results are provided in Table S7.

### Enriched GO processes in ParkinTAP and Related PD

To identify and prioritize biologically relevant ParkinTAP candidates, we compared the GO biological process enrichment results between ParkinTAP and RelatedPD and identified 19 GO terms that were significantly enriched in both datasets (classic score P≤10^−3^) (Table 3). Of these, five processes were significantly enriched with both classic and LEA scores (P≤10^−3^), and ParkinTAP candidates annotated to these five processes (or their child processes) are identified in Table S3 (columns GOComp; response to unfolded protein, mitochondrion organization, intracellular transport, establishment of localization in cell, cellular protein complex assembly). In addition, three of the 19 processes showed a significant LEA score (P≤10^−3^) in ParkinTAP: “protein folding”, “cellular macromolecular complex assembly”, and “cellular metabolic process”. ParkinTAP candidate proteins annotated to protein folding and descendant processes are also identified in Table S3 (column GOComp; protein folding).

**Table 3:** GO biological processes enriched in ParkinTAP and RelatedPD datasets.

	GO ID	GO Term	Classic score (P≤10^−3^)	LEA score (P≤10^−3^)	ParkinTAP LEA score (P≤10^−3^)*
1	GO:0071844	cellular component assembly at cellular level	√		
2	GO:0071842	cellular component organization at cellular level	√		
3	GO:0071840	cellular component organization or biogenesis	√		
4	GO:0071841	cellular component organization or biogenesis at cellular level	√		
5	GO:0006986	response to unfolded protein	√	√	
6	GO:0019538	protein metabolic process	√		
7	GO:0022607	cellular component assembly	√		
8	GO:0044267	cellular protein metabolic process	√		
9	GO:0016043	cellular component organization	√		
10	GO:0007005	mitochondrion organization	√	√	
11	GO:0046907	intracellular transport	√	√	
12	GO:0008152	metabolic process	√		
13	GO:0051649	establishment of localization in cell	√	√	
14	GO:0006457	protein folding	√		√
15	GO:0043623	cellular protein complex assembly	√	√	
16	GO:0035966	response to topologically incorrect protein	√		
17	GO:0034622	cellular macromolecular complex assembly	√		√
18	GO:0044237	cellular metabolic process	√		√
19	GO:0044085	cellular component biogenesis	√		

* ParkinTAP LEA score P≤10^−3^, RelatedPD LEA score p>10^−3.^

In addition, out of the significantly enriched GO terms in RelatedPD, we selected a set of five representative terms (GOSlimPD) (Table 4) and identified a total of 50 ParkinTAP proteins that were annotated to any of these five terms or to their descendants with a more specific annotation (Table 2 and Table S3; column GOSlimPD).

**Table 4:** Representative GO terms for monogenic parkinsonism (GOSlimPD).

GO ID	GO Term
GO:0008219	cell death
GO:0031396	regulation of protein ubiquitination
GO:0006950	response to stress
GO:0007005	mitochondrion organization
GO:0006914	autophagy

### Analysis of functional relationships

**Figure 2:**
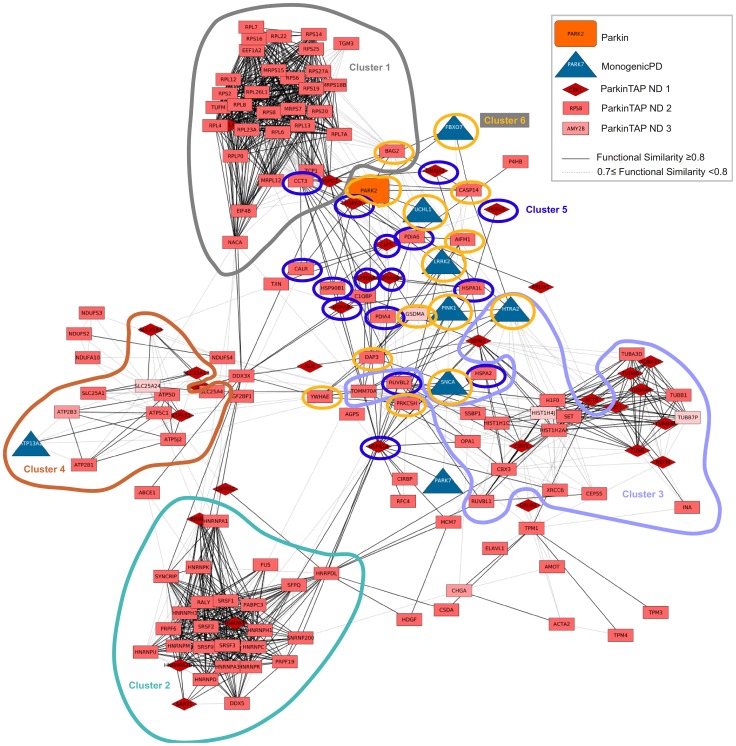
Functional similarity network FunSimPDsub. This network includes ParkinTAP candidates and MonogenicPD proteins with a functional similarity score ≥0.7; six significant clusters were identified (P≤0.05).

To further prioritize ParkinTAP candidates, we assessed the functional similarity between the candidate proteins and the proteins included in MonogenicPD. The FunSimPDsub network (Figure 2) represents the functional relationships between the ParkinTAP and MonogenicPD proteins that showed a functional similarity score ≥0.7. It includes 211 proteins, 157 of them in a single connected component including 149 ParkinTAP candidates. The remaining 54 candidate proteins are not functionally similar (functional similarity score ≥0.7) to any other protein in FunSimPDsub. In this network, six significant protein clusters (P≤0.05) were identified, and GO enrichment analysis was performed for the proteins included in each cluster. Cluster 1 proteins are mainly involved in RNA processing and translation, cluster 2 proteins are involved in transcription, RNA processing and splicing, cluster 3 represents processes related to complex assembly, protein folding, mitochondrion organization, and cytoskeleton-dependent intracellular transport, proteins in cluster 4 are involved in mitochondrial processes, like mitochondrial transport, mitochondrial ATP synthesis, and respiratory electron transport chain, cluster 5 proteins are involved in protein folding, and the over-represented processes in cluster 6, which contains most MonogenicPD proteins, are related to programmed cell death and mitochondrion organization. Detailed enrichment results for the six protein clusters are provided in Table S8.

**Figure 3:**
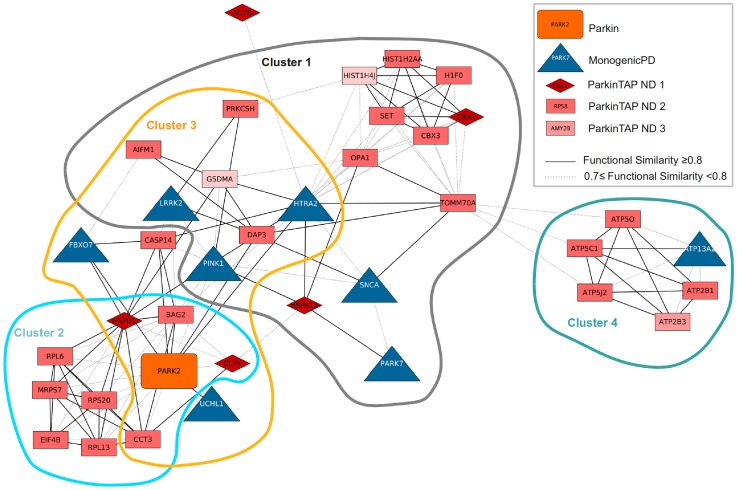
Functional similarity network FunSimPD_ND1. This network consists of ParkinTAP candidates that are functionally related to MonogenicPD proteins (functional similarity score ≥0.7); four significant clusters were identified (P≤0.05).

**Figure 4:**
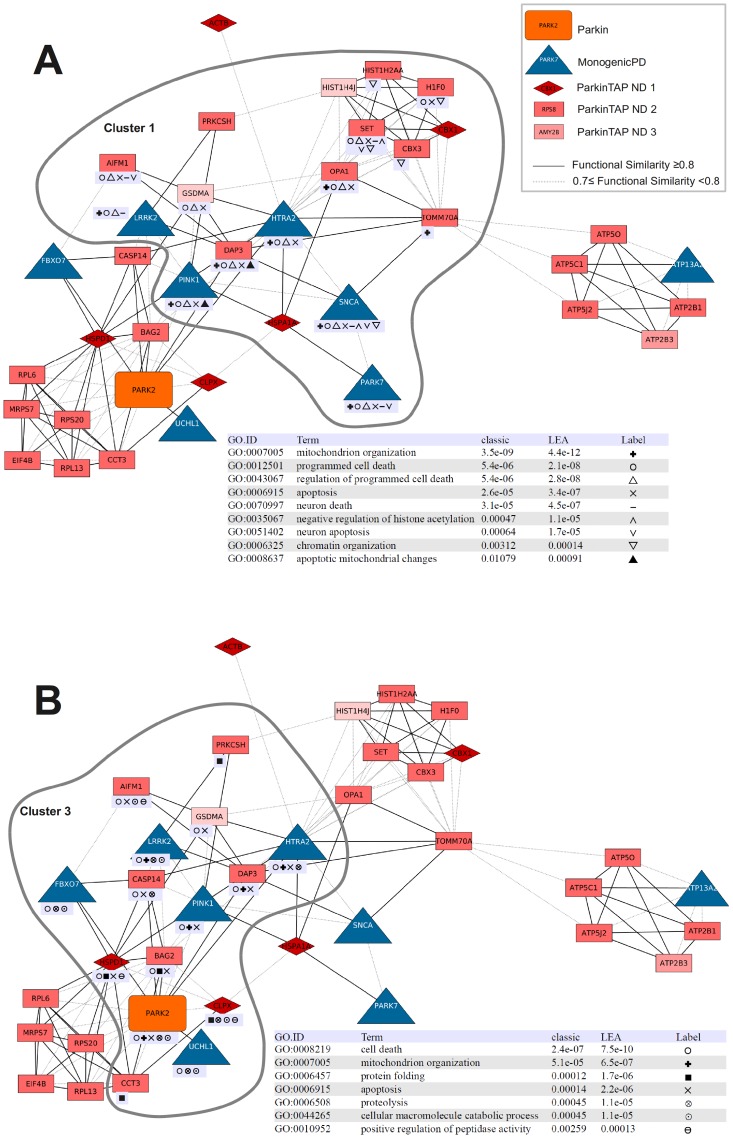
FunSimPD_ND1 clusters 1 (A) and 3 (B) with the enriched GO processes listed in the inserted tables. Proteins annotated to enriched processes are marked in the networks with symbols listed in the GO enrichment tables. To remove redundancy, enriched processes that are descendants of listed processes or that map only to MonogenicPD proteins were excluded.

To better investigate which ParkinTAP candidates are functionally related to MonogenicPD proteins, we generated the subnetwork FunSimPD_ND1 consisting of only MonogenicPD proteins and the ParkinTAP candidates that are functionally related to them (functional similarity score ≥0.7) (Figure 3). FunSimPD_ND1 includes 38 proteins in a single connected component, which were again grouped into four significant clusters (P≤0.05) with most MonogenicPD proteins contained in clusters 1 and 3. In cluster 1 of this network, biological processes related to programmed cell death and mitochondrion organization are enriched (Figure 4A), cluster 2 proteins are mostly involved in translation and protein folding, cluster 3 proteins are enriched in processes like programmed cell death, mitochondrion organization, protein folding and proteolysis (Figure 4B), and cluster 4 proteins are mainly involved in mitochondrial ATP synthesis.

### Comparison of ParkinTAP candidates to genetic interaction screens and GWAS data

In order to further assess the biological relevance of the Parkin-interacting proteins identified in our study, we compared our candidate dataset with the results of a recently published genetic screen for modifiers of *Parkin* and *PINK1* mutant phenotypes in *Drosophila* [26]. From this screen, 127 cytological regions were identified that enhanced or suppressed the *Parkin* wing-posture phenotype or caused lethality prior to adult stage. In these cytological regions, 5,420 human orthologues were annotated and an overlap of 94 proteins with our dataset of candidate Parkin-interacting proteins was identified (P = 6.0×10^−12^ relative to the total number of human genes and P = 1.5×10^−4^ relative to the total number of fly genes) (Table 2 and Table S3; column ParkinGS). The same analysis was performed for the *PINK1* mutant phenotypes, where 97 cytological regions were identified that affected the *PINK1* wing-posture phenotype or reduced fly viability [26]. These regions were mapped to 4,163 human orthologues, which overlap with 76 ParkinTAP candidates (P = 2.4×10^−10^ and P = 2.2×10^−4^ relative to human genes or fly genes, respectively) (Table 2 and Table S3; column Pink1GS).

The ParkinTAP candidates were also compared to human GWAS results for PD to look for evidence of potential association signals in or around the genes encoding these candidate proteins. Comparison to the PDGene database [27] resulted in only one gene overlap (*LMNA*) within a distance of 50 kb of the listed genetic variants. A variant within this gene has been associated with PD in a recent GWA meta-analysis (P = 2.4×10^−6^) [28].

### Prioritization of candidate proteins

A set of criteria was defined to assign a prioritization score (selection level) for each candidate protein, which is listed in the last column of Table 2 (for candidates with selection level 0–7) and Table S3 (for all candidates). The selection levels range from lowest priority level 8 to highest priority level 0. By default, the candidate proteins have a selection level 8; if they are annotated to PD-related processes, they are assigned to selection level 7, and if they either interact or are functionally similar to PD-related proteins, the selection level is 6. Candidates with the selection levels 3, 4, and 5 interact or are functionally similar to PD-related proteins, and in addition match candidates from the PINK1TAP screen, or are annotated to PD-related processes, overlap with the Parkin/PINK1 fly genetic screen or do not interact with many proteins (and therefore tend to make unique/specific interactions). If the candidates both, interact and are functionally similar to PD-related proteins, they are prioritized with selection level 2. Candidates that interact with more than one PD-related protein are assigned to top rank selection level 1. Selection level 0 is reserved to candidates that have been reported to interact with Parkin in HNet. The criteria are outlined in the Supporting Information and visualized in an overview graph (Figure S2).

### Comparison of ParkinTAP candidates to Endeavour

Using Endeavour [29], an independent gene prioritization was performed. In general, the genes prioritized with Endeavour are also top ranking according to the selection levels described in the previous section. In total, eight genes have an Endeavour prioritization score <0.01, and seven of these genes also have a selection level ≤5. The Endeavour results and the corresponding selection levels according to our prioritization are provided in Table S9.

### Comparison of ParkinTAP candidates to a dataset of Parkin substrates and interactors

A recent study provided a systematic identification of Parkin-dependent ubiquitylation targets and interacting proteins [30]. Eight out of 155 proteins reported to interact with Parkin in this study (weighted and normalized D-scores ≥1.0) match the ParkinTAP candidates listed in Table S3. Parkin is one of the matching proteins, three other matching candidates have a selection level of 3 (TOMM70A, TUBA1C, TUBA4A), the remaining four candidates have selection levels 7 and 8 (SLC25A4, RPS27A, TUBB7P, EEF1A2). One of these matching candidates (RPS27A) was prioritized also by Endeavour. The overlap between the ParkinTAP candidates and the interaction results from this study is statistically significant (Fisher's exact test P<1×10^−3^). In addition, seven out of 99 Parkin ubiquitylation targets (class 1 results) are also included in the ParkinTAP candidates from our study. Parkin is again one of the matching candidates, two additional matching candidates have selection level 0 (HSPA8, HSPA1A), other four have selection level 3 (TOMM70A, HSP90AB1) or selection levels 6 and 7 (HNRNPF, YWHAE). The overlap is again statistically significant (P<1×10^−3^).

### Co-immunoprecipitation

**Figure 5:**
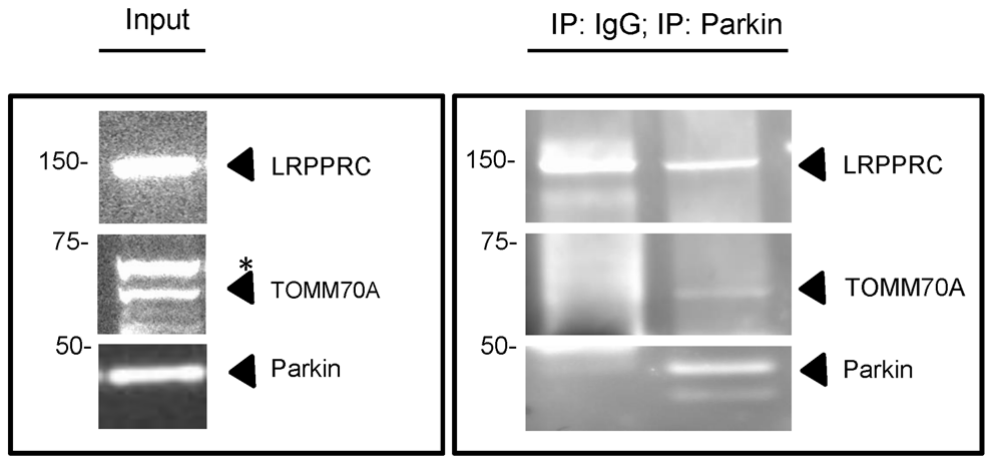
Co-immunoprecipitation assays for Parkin and candidate binding proteins. Extracts from untransfected HEK293T cells were subjected to immunoprecipitation with anti-Parkin or control IgG antibodies, followed by Western Blot of input and immunoprecipitation (IP) fractions with antibodies against LRPPRC and TOMM70A. The asterisk indicates a non-specific band.

For validation of candidate Parkin-binding proteins, we performed co-immunoprecipitation experiments from HEK293T cells for the two candidates shown in Figures 1A and 1B (LRPPRC and TOMM70A). Figure 5 shows the results of the co-immunoprecipitation using antibodies raised against the two candidate Parkin-interacting proteins. We observed co-immunoprecipitation with TOMM70A, whereas the interaction with LRPPRC seems to be non-specific as the Western Blot shows a band on the same height also in the negative control (control IgG antibodies).

## Discussion

Although the *Parkin* gene was identified 15 years ago [31], the multiple functions of this protein and the precise mechanisms by which it exerts its protective effect remain the subject of intense investigations. An important step in understanding the various functions of Parkin is placing it in a network of biochemical pathways, as the breakdown of these cellular pathways or processes, in which a group of proteins work together, may result in Parkin-associated pathology. To understand such network perturbations, it is necessary to systematically explore the complex interaction network in which the Parkin-binding proteins are interconnected.

In this study we identified 203 candidate Parkin-binding proteins using TAP/MS proteomic screens. The interactions between these proteins were investigated within HNet, and most of them were part of a single connected component. Whereas only three candidate proteins are known Parkin-binding proteins according to HNet (DNAJA1, HSPA1A, HSPA8) [23], 164 interact with Parkin through one intermediate protein, and 40 interact with one or two other proteins known to be involved in monogenic parkinsonism, which suggests that they might function together in a PD-specific pathway. This is supported by the finding that proteins linked to the same disease have a high propensity to interact with each other and that proteins in a close network-based vicinity to a disease-related protein can therefore be expected to play a role in the same disease-related process [32].

The biological processes enriched in the identified candidate proteins were compared to the processes enriched in the proteins causing monogenic forms of parkinsonism. Several processes, like protein folding, response to unfolded proteins, mitochondrion organization, and cellular protein complex assembly were found to be significantly enriched in both datasets. Also in the pathway analysis, the protein-folding pathway is enriched in the candidate proteins, and protein processing in the endoplasmic reticulum is significantly enriched both in the candidates and in the PD-related proteins.

By analyzing the functional similarity between the candidate proteins and the PD-related proteins, we identified six functional groups related to RNA processing, complex assembly, protein folding, intracellular transport, mitochondrial transport and ATP synthesis, and programmed cell death. These six protein clusters contained 149 candidate Parkin-binding proteins in total. Whereas most of the candidates are functionally similar amongst themselves, in cluster 6 the candidates are all functionally related to the PD-related proteins, with the only exception of YWHAE (Figure 2). A sub-network containing only the candidate proteins that are functionally similar to the PD-related proteins includes 37 proteins in total and 29 candidate Parkin-binding proteins (Figure 3). Twenty-eight candidates fall into four clusters of enriched GO processes that cover the functions of the known PD proteins, including the various known functions of Parkin. Among these processes we have identified several proteins involved in cell death: HSPD1, CASP14, H1F0, DAP3, AIFM1, GSDMA, SET, OPA1, and BAG2. In addition, TOMM70A, DAP3 and OPA1 are involved in mitochondrion organization, and CCT3, CLPX1, HSPD1, PRKCSH and BAG2 are related to protein folding. Of the 29 candidates that are functionally similar to the PD-related proteins, 13 were also identified in a genetic screen as modifiers of *Parkin* and *PINK1* mutant functions in *Drosophila* (three additional candidates were identified only as *Parkin* modifiers) [26], providing further evidence for the biological relevance of the interactions (Table 2).

The diverse functions of the Parkin protein partners reported here are consistent with the functional diversity of the pathogenic processes associated with *Parkin*-linked parkinsonism. Parkin is localized in the endoplasmic reticulum, the Golgi apparatus, the outer nuclear membrane, synaptic vesicles [33], [34], and the outer mitochondrial membrane [35], and there is a large body of evidence showing that Parkin can interfere with a diverse range of cellular processes and pathways. Like other E3 ubiquitin ligases, it is a component of the ubiquitin proteasome system (UPS), a main cellular pathway that promotes removal of damaged or misfolded proteins [36], it is involved in signal transduction, protein and membrane trafficking and transcriptional regulation [37]–[39], replication and transcription of mitochondrial DNA [35], mitophagy [13], neuroprotection [40], and apoptosis [41].

Parkin expression has been reported to protect cells against multiple forms of stress [42], but although the exact mechanism of this prosurvival function remains elusive, accumulating evidence exists that it involves inhibition of programmed cell death (apoptosis). Two recent studies identified Bax and the mitochondrial pro-apoptotic protein ARTS as Parkin substrates that both might contribute to the anti-apoptotic effect of Parkin [43], [44]. In our study, we identified novel associations between Parkin and several proteins involved in cell death processes. An interaction of Parkin with one of them, OPA1, is supported by the observation that inactivation of OPA1, which promotes mitochondrial fusion, rescues the phenotypes of cell death, muscle degeneration, and mitochondrial abnormalities in *Parkin* and *PINK1* mutants in *Drosophila* [11]. DAP3, another candidate protein involved in cell death, mediates mitochondrial fragmentation, probably reflecting its role in mitochondrial fission [45]. Both proteins might have a role in cell death-associated changes in mitochondrial morphology mediated by Parkin. TOMM70A, which encodes a component of a translocase complex of the outer mitochondrial membrane involved in the import of mitochondrial precursor proteins [46], has been associated recently to Parkin as it is degraded by the UPS after translocation of Parkin to mitochondria [47]. LRPPRC, which was identified already in a proteomic analysis of Parkin interactors [48], might be involved in mitophagic initiation, maturation, trafficking, and lysosomal clearance through its interaction with the MAP1S protein [49]. Other Parkin-binding proteins, like HSPD1 and CLPX, are involved in protein folding and response to unfolded proteins. HSPD1 is one of the most important components of the protein folding system within the mitochondrial matrix [50], and CLPX functions in substrate degradation [51].

*In vitro* derived TAP results contain false positive interactions and do not represent all binding proteins. Although the two sequential purification steps of the TAP method largely reduce the background resulting from non-specific protein binding compared to a single purification step, these contaminants cannot be completely removed. A limitation of the TAP/MS approach, which preferentially detects interactions within a protein complex [52], is that it is not very powerful for the detection of transient interactions, such as between E3 ubiquitin ligases and their substrates. Therefore, the proteins identified in our study might more likely be Parkin-binding partners than Parkin substrates. In this respect, it is however reassuring that there is a statistically significant overlap between the ParkinTAP candidates from our study and the Parkin interactions and ubiquitylation targets reported in another recent study [30].

Furthermore, the purification step involving the Calmodulin-binding peptide has proven to be problematic when many proteins interact with calmodulin in a calcium-dependent manner [53]. Candidate Parkin-binding proteins that bind also to Calmodulin might therefore be potential TAP artifacts (Table 2 and Table S3; CalmodulinIP). Also, the binding peptides might disturb the function of the tagged proteins. However, similar to untagged Parkin, TAP-tagged Parkin translocated to depolarized mitochondria and induced their removal, indicating that the tag did not interfere with Parkin-mediated mitophagy (data not shown).

Protein interaction data generated by any method need to be confirmed through experimental validation, like co-immunoprecipitation assays. For one of two candidate proteins tested, we were able to confirm a physical interaction with Parkin. However, whereas immunoprecipitation gives validation of the physical interaction of proteins, genetic screens in model organisms like *Drosophila melanogaster* provide additional information about the biological relevance of the interaction of candidate proteins and their putative role in genetic pathways related to PD. The utility of combining protein-interaction screening with genetic-interaction screening to validate protein-protein interaction data was shown in a screen for huntingtin-interacting proteins [54]. In addition, a simple comparative analysis between the candidate Parkin-binding proteins and the results from previously published GWAS was performed. A more extensive analysis will be the focus of future work, in particular by considering SNPs with P-values below the reported significance thresholds and by accounting for multiple genes that may be in the same linkage disequilibrium block as associated variants.

Prioritization of candidate binding proteins based on functional annotations might result in a “knowledge bias” towards well-characterized genes. In our study, this is partially countered by also considering previously reported protein interactions, which should not be affected by the same type of bias. However, a careful interpretation of the interactome results is necessary given the noise in the public data, in particular regarding complexes, where the exact interaction partners within the complex are unknown [55]. The network of protein interactions involving PD-related proteins and the candidate Parkin-binding proteins includes a relative large number of interactions within multi-protein complexes. This effect can be quantified by measuring the network density (ratio between the number of edges and the theoretical maximum number of edges). For example, the network density of the shortest path network SpNet is 3.6%. This relatively high network density reflects the nature of the many processes involved in PD, but it is also a result of the noise in the complex interaction data that is magnified as a result of the matrix expansion described in the Methods section. In this regard there have been some efforts to annotate the reported protein interactions with reliability scores [56]. Once such reliability scores are widely available, they can be used to filter unreliable interactions, which should result in less dense and noisy networks.

In summary, our study has identified novel candidate Parkin-binding proteins with diverse functions that can be associated to the many pathogenic processes of Parkin-linked parkinsonism. The functional diversity of the Parkin-binding proteins and their involvement in cell death processes, protein folding and response to unfolded proteins, the fission/fusion machinery, and the mitophagy pathway further reveals the diversity and complexity of Parkin function and confirms the large impact of Parkin on cellular physiology. Further studies are necessary to generate high quality, comprehensive interaction datasets for other PD proteins, which can be used to identify shared disease pathways and their components. Focusing not just on individual proteins but, on a network of proteins will prove essential to provide new targets for the development of therapeutic interventions.

## Methods

### Cell culture

In this study two cell lines were used: Human embryonic kidney cells (HEK293T, ATCC CRL-11268) and Human neuroblastoma cells (SH-SY5Y, ATCC CRL-2266). HEK293T were cultured in Dulbecco's modified Eagle's Medium (DMEM) supplemented with 10% fetal bovine serum and 1% penicillin-streptomycin (all Lonza). SH-SY5Y, ATCC CRL-2266 were cultured in DMEM-F:12 (Lonza) supplemented with 10% fetal bovine serum and 1% penicillin-streptomycin. Cells were maintained at 37°C in a saturated atmosphere containing 5% CO_2_. To dissipate the mitochondrial membrane potential, cells were treated with the potassium ionophore valinomycin (1 µM, Sigma) or the protonophore *m*-chlorophenylhydrazone (CCCP) (10 µM, Sigma).

### Mitochondrial preparation

Mitochondria were isolated from HEK293T and SH-SY5Y cells as previously described [57]. In brief, cells were harvested and homogenized in buffer containing 250 mM sucrose, 10 mM Tris, 1 mM EDTA, pH 7.4 containing protease and phosphatase inhibitors (Roche Diagnostics). In order to remove nuclei and unbroken cells, the homogenate was centrifuged twice at 1,500×g for 10 min. The supernatant containing intact mitochondria was transferred into a new tube and centrifuged at 8,400×g for 10 min. The resulting supernatant (“cytosolic fraction”) was centrifuged once again to obtain a purer fraction (8,400×g for 10 min), whereas the mitochondria-enriched pellet (“mitochondrial fraction”) was washed once with the buffer described above and centrifuged at 8,400×g for 10 min.

### Tandem Affinity Purification

To identify Parkin-interacting proteins, the InterPlay Mammalian Tandem Affinity Purification (TAP) System was used according to the manufacturer's instructions (Agilent Technologies). TAP was performed for whole cell lysates of HEK293T cells as well as for mitochondrial and cytosolic fractions of treated and not treated HEK293T and SH-SY5Y cells. In brief, full-length *Parkin* cDNA was cloned downstream of the multiple cloning site into the pCTAP expression vector, which encodes two different affinity purification tags (a streptavidin and a calmodulin binding peptide). Subsequently, 10^8^ HEK293T and SH-SY5Y cells were transiently transfected with the pCTAP-*Parkin* vector using the CaPO_4_ method [58]. Whole cell and mitochondrial pellets were resuspended in lysis buffer supplemented with protease and phosphatase inhibitors, whereas the cytosolic fraction was already dissolved in the buffer used for the mitochondrial preparation. Next, 2 mM EDTA, 10 mM β-mercaptoethanol, and the streptavidin resin were added to the cell lysate and incubated at 4°C while rotating for 2 h. The resin was collected by centrifugation (1,500×g, 5 min), washed twice and incubated with biotin-containing streptavidin elution buffer for 30 min at 4°C to elute the protein complexes. As a second purification step, calmodulin resin and a calcium containing buffer were added to the eluate and the mixture was incubated at 4°C on a rotator for 2 h. The resin was collected by centrifugation (1,500×g, 5 min) and washed twice. Bound protein complexes were eluted using the EDTA-containing calmodulin elution buffer for 30 min at 4°C. The final eluate was concentrated using the ProteoExtract® Protein Precipitation Kit (Merck Millipore), and the precipitate was sent for identification of the unknown proteins to the Taplin Biological Mass Spectrometry Facility (Harvard Medical School, Boston, USA). According to their guidelines, only proteins with two or more peptide matches with the respective protein in the UniProtKB/Swiss-Prot database are confidently identified from the sample.

### Bioinformatic analyses

#### Protein identifiers

Ensembl Biomart 64 [59] was used to map between Entrez Gene ID, HGNC gene symbols and UniProtKB accession numbers. *Drosophila melanogaster* genes were mapped to human orthologous using InParanoid 7.0 [60]. All comparisons between datasets were made using Entrez Gene IDs, except for the comparison to the genetic interaction screen results in *Drosophila*, which was performed with Ensembl Gene ID. In the following, the HGNC gene symbols are used to identify both genes and their encoded proteins, according to the context.

#### Protein-protein interactions

As a source of known protein-protein interactions, iRefIndex 9.0 was used, which combines protein interaction data from multiple primary resources [55]. There are two types of interactions: binary interactions, which involve two interactors, and complex interactions, which are characterized by more than two interactors and where the pairwise physical contacts are not specified. Both types of interactions were used to build the network models. Protein complexes were expanded using the matrix expansion model, where pairwise interactions are assigned between all interactors within a complex. Data were filtered to exclude predicted interactions, in particular interactions, for which the detection method contained “predicted”, “interologs mapping” or “confirmational text mining”. The resulting network was labeled “HNet”.

From this network, a shortest path network (SpNet) was derived by selecting all proteins and interactions within the shortest paths between the ParkinTAP candidates identified in our study and the MonogenicPD proteins [20], the known Parkin interactors (ParkinIP) and the MonogenicPD interactors (MonogenicPDIP) (see protein datasets in Table 1; the proteins contained in the datasets are included in Table S1). DAPPLE, which uses InWeb, a curated interaction network [61], was used to assess the statistical significance of the connectivity within the ParkinTAP candidates and the MonogenicPD proteins.

#### Pathway enrichment analysis

Pathway enrichment analysis was performed with ConsensusPathDB-human release 23 [62], which integrates different databases of human biological processes, metabolic and signaling pathways and protein interactions. Pathway enrichment analysis was performed for a given protein dataset by computing a P-value for each pathway according to the hypergeometric test and corrected for multiple testing using the false discovery rate method (FDR). The procedure is described in ConsensusPathDB as “over-representation analysis” on pathway-based protein datasets.

#### Gene ontology enrichment analysis

Functional annotation was provided by the Gene ontology (GO) project [63]. GO enrichment analysis was performed using topGO [64], a method that takes into account dependencies between GO terms resulting from the GO graph topology. Version 2.6.0 of topGO was used, as provided with Bioconductor version 2.9. The analysis was restricted to GO biological processes, and two different GO enrichment scores were computed. The “classic” score is based on the standard Fisher's exact test and is a P-value corrected for multiple testing by FDR, but it does not take into account the GO graph structure and the children/parent dependencies between GO terms. The “LEA” score is locally adjusted for the dependencies between GO terms, where more generic terms are down-weighted versus descendant (more specific) GO terms. The GO processes are sorted by LEA score (P≤10^−3^), and the multiple test corrected classic score is used to confirm the significance of the GO terms selected with LEA.

#### Functional similarity

Functional similarity between proteins was computed using FunSimMat release 4.2 as previously described [65], [66]. In particular, FunSimMat computes a semantic measure of functional similarity based on the GO annotation obtained from the Gene Ontology Annotation database [67]. The analysis was restricted to biological processes. Each protein was identified by the corresponding Entrez Gene ID, which was mapped to UniProtKB accession numbers. In many cases, multiple UniProtKB accession numbers were mapped to a single initial gene ID (mapping is many to many), and in these cases the functional similarity corresponded to the maximum functional similarity obtained for any of the UniProtKB entries. A functional similarity network (FunSimPD) was generated, where nodes correspond to proteins from ParkinTAP, MonogenicPD and MonogenicPDIP datasets. Proteins were connected by edges, if their functional similarity score was ≥0.7. From FunSimPD two subnetworks were extracted for detailed analysis (FunSimPDsub, FunSimPD_ND1), and distinct groups of functionally related proteins were identified by graph clustering.

#### GO Slims

QuickGO [68] was used to identify proteins from a given dataset that have been annotated with a GO term or children from a set of specific GO terms (GO Slim).

#### Network analysis and visualization

Cytoscape 2.8.1 was used for network visualization of protein-protein interactions and functional relationships [69]. Clustering was performed with the Cyotscape plugin ClusterONE, which can identify densely connected overlapping regions within networks [70]. Edges weighted according to functional similarity score were used for clustering the functional similarity networks.

#### Endeavour gene prioritization

An independent gene prioritization of the candidate Parkin-binding proteins was performed with Endeavour [29], an established prioritization tool. Ensembl gene IDs were provided as input, the MonogenicPD dataset was used for training, and all data sources available in Endeavour were used for prioritization.

### Co-immunoprecipitation and Western Blot

HEK293T cells were washed with ice-cold PBS, removed with a scraper, and resuspended in lysis buffer (150 mM NaCl, 50 mm Tris-HCl, 1% Triton X-100, and 0.1% SDS, pH 7.6). After incubation for 30 min at 4°C, insoluble material was removed by centrifugation at 14,000×g for 10 min. Protein concentrations were determined using the Bio-Rad *DC* Protein assay (Bio-Rad). The sample was precleared by incubation with protein A Agarose beads (Roche Diagnostics) for 30 min at 4°C. The beads were removed by centrifugation and the samples were incubated overnight at 4° with rabbit anti-Parkin (Abcam, ab15954) or control IgG (purified rabbit IgG, Millipore). The antigen-antibody complexes were captured by addition of protein A Agarose for 2 h at 4°C and washed three times with wash buffer (150 mM NaCl, 50 mM Tris-HCl, 1% Igepal, pH 7.6). Proteins were released from the beads by heating at 95°C for 5 min in 4x Sample buffer containing DTT (Life Technologies), followed by SDS-PAGE, blotting onto nitrocellulose membranes, and incubation with anti-TOMM70A (Abcam, ab135602), LRPPRC (Abcam, ab97505), and Parkin (Cell Signaling, #4211) antibodies. Immunoreaction was visualized using the SuperSignal West Dura Chemiluminescence Westernblot Substrate (Thermo Scientific).

## Supporting Information

Figure S1 I**nteraction networks for ParkinTAP candidate proteins CLPX (A), PRKCSH (B), DAP3 (C), and CALU (D).** Proteins are represented as nodes and interactions as edges; the edges are drawn as solid and dashed lines for binary and complex interactions, respectively. Interactions to the selected candidate proteins are represented by thicker edges. ParkinTAP ND X are ParkinTAP candidates at network distance X of MonogenicPD, where ParkinTAP ND 1 are direct MonogenicPD interactors.
(TIF)

Figure S2 **Overview of criteria for the definition of the selection levels.** The different datasets are labeled according to the legend of Table S3. “L” stands for selection level.
(TIF)

Table S1 **Proteins contained in the datasets listed in** **Table 1** **.**
(TSV)

Table S2 **ParkinTAP proteins that do not interact with the other candidate proteins.**
(TSV)

Table S3 **Summary table for all ParkinTAP candidate proteins.**
(TSV)

Table S4 **ConsensusPathDB enrichment results for the RelatedPD dataset.**
(TSV)

Table S5 **GO enrichment results for the RelatedPD dataset.**
(TSV)

Table S6 **ConsensusPathDB enrichment results for ParkinTAP.**
(TSV)

Table S7 **GO enrichment results for ParkinTAP.**
(TSV)

Table S8 **GO enrichment results for FunSimPDsub clusters.**
(PDF)

Table S9 **Gene prioritization results for the ParkinTAP candidates using Endeavour, including the corresponding selection levels according to our prioritization.**
(TSV)

Text S1 **Legend Table S3. Selection level pseudocode.**
(DOCX)
