# Supplementary figures and images for: Profiling of Parkin-Binding Partners Using Tandem Affinity Purification
Source: PLoS One. 2013 Nov 11;8(11):e78648. doi: 10.1371/journal.pone.0078648 (PMC3823883; doi:10.1371/journal.pone.0078648)

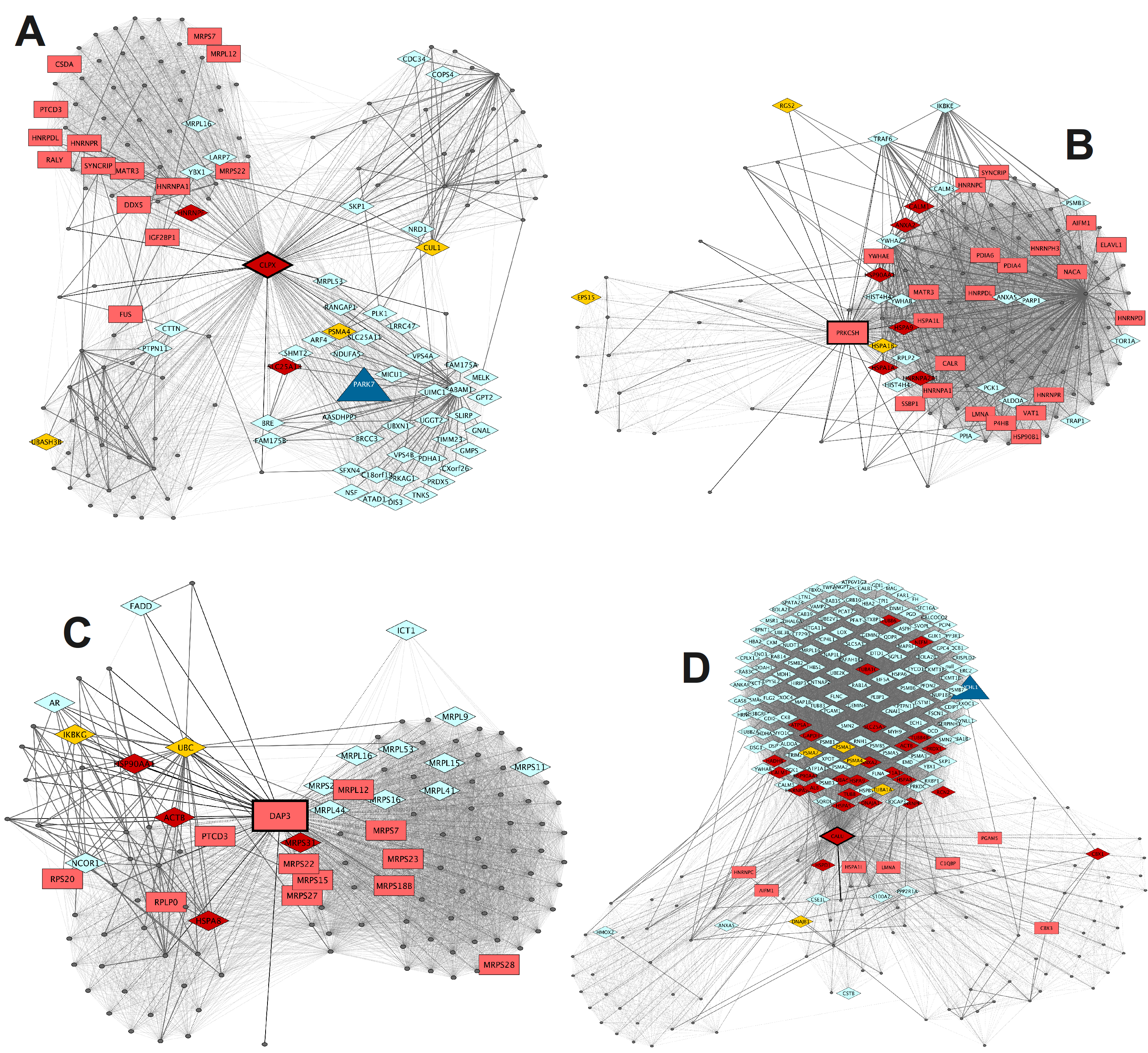

Supplement: Figure S1 — Interaction networks for ParkinTAP candidate proteins CLPX (A), PRKCSH (B), DAP3 (C), and CALU (D). Proteins are represented as nodes and interactions as edges; the edges are drawn as solid and dashed lines for binary and complex interactions, respectively. Interactions to the selected candidate proteins are represented by thicker edges. ParkinTAP ND X are ParkinTAP candidates at network distance X of MonogenicPD, where ParkinTAP ND 1 are direct MonogenicPD interactors. (TIF) [file pone.0078648.s001.tif]

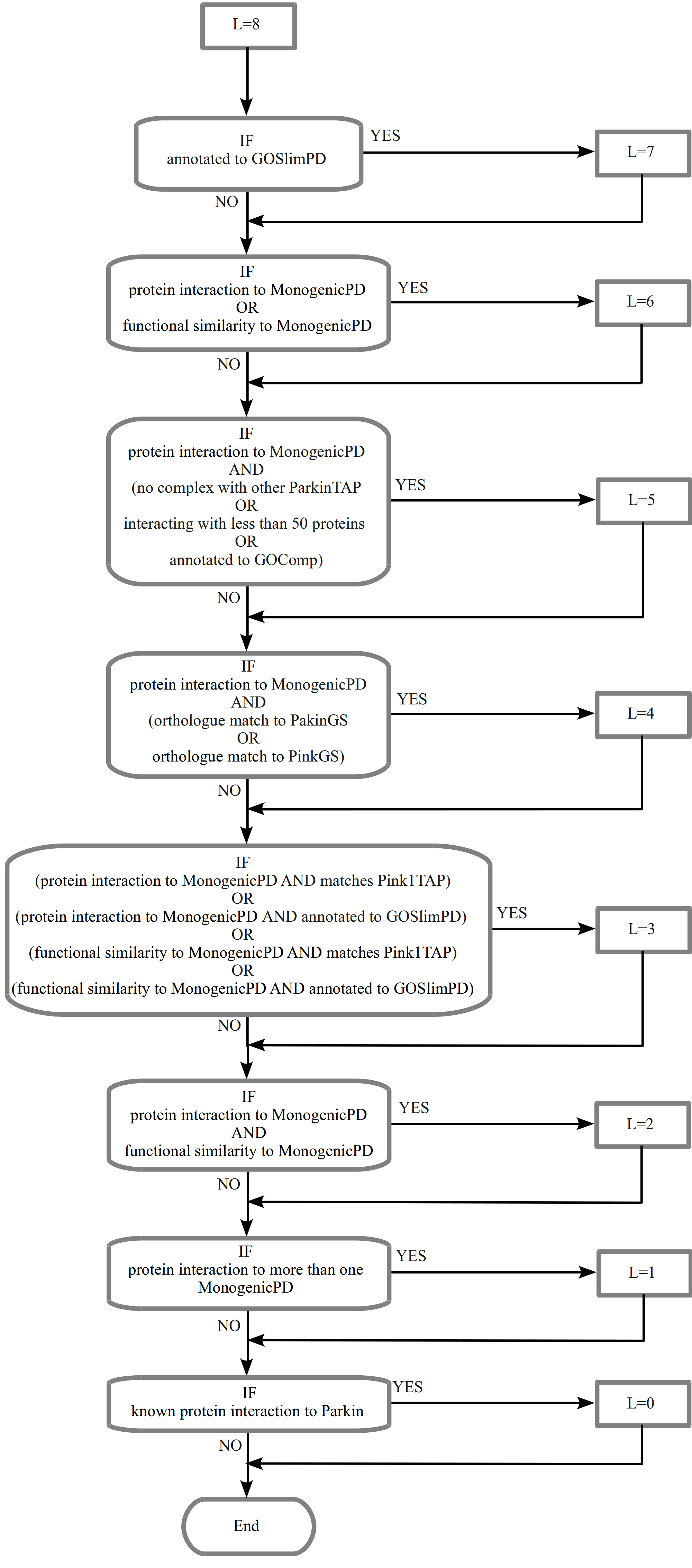

Supplement: Figure S2 — Overview of criteria for the definition of the selection levels. The different datasets are labeled according to the legend of Table S3. “L” stands for selection level. (TIF) [file pone.0078648.s002.tif]
