# Supplementary material for: Profiling of Parkin-Binding Partners Using Tandem Affinity Purification
Source: PLoS One. 2013 Nov 11;8(11):e78648. doi: 10.1371/journal.pone.0078648 (PMC3823883; doi:10.1371/journal.pone.0078648)
Supplement: Table S8 — GO enrichment results for FunSimPDsub clusters. (PDF) [file pone.0078648.s010.pdf]

Table S7

Cluster 1

Rank	GO.ID	Term	Annotated	Significant	Expected	Rank in classic	classic	LEA
1	GO:0006414	translational elongation	104	22	0.24	1	< 1e-30	< 1e-30
2	GO:0006415	translational termination	93	20	0.22	3	< 1e-30	< 1e-30
3	GO:0031018	endocrine pancreas development	124	20	0.29	4	< 1e-30	< 1e-30
4	GO:0019083	viral transcription	146	20	0.34	5	3.3e-30	< 1e-30
5	GO:0016071	mRNA metabolic process	608	21	1.42	23	1.1e-19	4.4e-21
6	GO:0042274	ribosomal small subunit biogenesis	18	6	0.04	31	3.4e-11	1.9e-12
7	GO:0006412	translation	426	29	1	2	< 1e-30	1.9e-09
8	GO:0006364	rRNA processing	99	6	0.23	48	1.2e-06	9.9e-08
9	GO:0000028	ribosomal small subunit assembly	6	3	0.01	51	2.6e-06	2.3e-07
10	GO:0030490	maturation of SSU-rRNA	7	2	0.02	64	0.00098	0.00011
11	GO:0044267	cellular protein metabolic process	2809	34	6.57	17	1.5e-23	0.00014
12	GO:0006458	'de novo' protein folding	52	3	0.12	65	0.00208	0.00024

Cluster 2

Rank	GO.ID	Term	Annotated	Significant	Expected	Rank in classic	classic	LEA
1	GO:0000398	nuclear mRNA splicing, via spliceosome	201	26	0.39	1	< 1e-30	< 1e-30
2	GO:0006405	RNA export from nucleus	68	6	0.13	17	4.2e-08	2.9e-09
3	GO:0000245	spliceosome assembly	37	5	0.07	22	9.7e-08	7.6e-09
4	GO:0006369	termination of RNA polymerase II transcription	44	5	0.08	24	2.1e-07	1.9e-08
5	GO:0051028	mRNA transport	110	6	0.21	29	5.2e-07	5.4e-08
6	GO:0006406	mRNA export from nucleus	59	5	0.11	30	7.7e-07	8.5e-08
7	GO:0031124	mRNA 3'-end processing	74	5	0.14	32	2.4e-06	2.7e-07
8	GO:0043484	regulation of RNA splicing	49	4	0.09	36	1.7e-05	2.2e-06
9	GO:0000380	alternative nuclear mRNA splicing, via spliceosome	22	3	0.04	39	6.6e-05	9.6e-06
10	GO:0048255	mRNA stabilization	22	3	0.04	40	6.6e-05	9.6e-06
11	GO:0070934	CRD-mediated mRNA stabilization	5	2	0.01	46	0.00022	3.6e-05
12	GO:0045292	nuclear mRNA cis splicing, via spliceosome	6	2	0.01	47	0.00032	5.3e-05
13	GO:0006376	mRNA splice site selection	17	2	0.03	49	0.00275	0.00048

Cluster 3

Rank	GO.ID	Term	Annotated	Significant	Expected	Rank in classic	classic	LEA
1	GO:0071842	cellular component organization at cellular level	2803	24	4.82	1	5.7e-14	1.3e-16
2	GO:0034622	cellular macromolecular complex assembly	456	14	0.78	3	3.5e-13	2.4e-15
3	GO:0007018	microtubule-based movement	128	10	0.22	4	6.2e-13	5.7e-15
4	GO:0051084	'de novo' posttranslational protein folding	47	7	0.08	8	5.8e-11	1.1e-12
5	GO:0006996	organelle organization	1768	18	3.04	12	2.2e-10	6.4e-12
6	GO:0051258	protein polymerization	126	8	0.22	14	7.6e-10	2.4e-11
7	GO:0006334	nucleosome assembly	102	6	0.18	20	3.6e-07	1.6e-08
8	GO:0030705	cytoskeleton-dependent intracellular transport	36	4	0.06	28	5.6e-06	3.9e-07
9	GO:0060052	neurofilament cytoskeleton organization	6	2	0.01	36	0.00051	4.2e-05
10	GO:0043968	histone H2A acetylation	12	2	0.02	37	0.00218	0.00019
11	GO:0007005	mitochondrion organization	175	4	0.3	38	0.00242	0.00021
12	GO:0070925	organelle assembly	76	3	0.13	39	0.00320	0.00029
13	GO:0008088	axon cargo transport	15	2	0.03	40	0.00320	0.00029
14	GO:0006338	chromatin remodeling	84	3	0.14	41	0.00415	0.00039
15	GO:0034080	CenH3-containing nucleosome assembly at centromere	22	2	0.04	46	0.00571	0.00064
16	GO:0022402	cell cycle process	938	7	1.61	54	0.00631	0.00078
17	GO:0042267	natural killer cell mediated cytotoxicity	27	2	0.05	57	0.00729	0.00097

Cluster 4

Rank	GO.ID	Term	Annotated	Significant	Expected	Rank in classic	classic	LEA
1	GO:0006754	ATP biosynthetic process	70	8	0.06	1	1.8e-14	9.3e-17
2	GO:0006839	mitochondrial transport	85	6	0.07	13	5.5e-10	3.0e-11
3	GO:0006811	ion transport	913	10	0.75	14	8.6e-10	5.3e-11
4	GO:0055085	transmembrane transport	934	10	0.77	16	1.0e-09	6.7e-11
5	GO:0042776	mitochondrial ATP synthesis coupled proton transport	15	4	0.01	18	4.8e-09	3.6e-10
6	GO:0006091	generation of precursor metabolites and energy	424	7	0.35	28	9.8e-08	1.2e-08
7	GO:0022904	respiratory electron transport chain	106	5	0.09	31	1.1e-07	1.4e-08
8	GO:0006812	cation transport	629	7	0.52	42	1.0e-06	1.8e-07
9	GO:0006200	ATP catabolic process	72	4	0.06	43	1.4e-06	2.7e-07
10	GO:0015711	organic anion transport	64	3	0.05	47	8.8e-05	1.7e-05
11	GO:0015813	L-glutamate transport	17	2	0.01	50	0.0004	8.4e-05
12	GO:0070588	calcium ion transmembrane transport	40	2	0.03	65	0.0017	0.00048
13	GO:0006094	gluconeogenesis	56	2	0.05	70	0.0032	0.00094

Cluster 5

Rank	GO.ID	Term	Annotated	Significant	Expected	Rank in classic	classic	LEA
1	GO:0006457	protein folding	213	12	0.22	1	1.8e-17	3.1e-20
2	GO:0006986	response to unfolded protein	68	7	0.07	2	6.5e-11	2.2e-13
3	GO:0006200	ATP catabolic process	72	4	0.07	6	7.1e-05	7.2e-07
4	GO:0045429	positive regulation of nitric oxide biosynthetic process	26	2	0.03	11	0.017	0.00032
5	GO:0051603	proteolysis involved in cellular protein catabolic process	371	4	0.38	12	0.019	0.00046
6	GO:0030433	ER-associated protein catabolic process	32	2	0.03	13	0.019	0.00048
7	GO:0051208	sequestering of calcium ion	39	2	0.04	14	0.019	0.00072

Cluster 6

Rank	GO.ID*	Term	Annotated	Significant	Expected	Rank in classic	classic	LEA
1	GO:0008219	cell death	1561	13	1.5	1	1.2e-09	3.0e-12
2	GO:0012501	programmed cell death	1424	11	1.37	3	5.3e-07	2.1e-09
3	GO:0043067	regulation of programmed cell death	1088	10	1.05	4	7.6e-07	4.0e-09
4	GO:0007005	mitochondrion organization	175	6	0.17	6	9.7e-07	7.7e-09
5	GO:0006915	apoptosis	1413	10	1.36	7	5.5e-06	5.1e-08
6	GO:0008344	adult locomotory behavior	47	4	0.05	8	7.9e-06	9.4e-08
7	GO:0042981	regulation of apoptosis	1079	9	1.04	9	7.9e-06	9.4e-08
8	GO:0043068	positive regulation of programmed cell death	548	7	0.53	10	2.1e-05	2.8e-07
9	GO:0006917	induction of apoptosis	380	6	0.37	13	4.3e-05	7.7e-07
10	GO:0001963	synaptic transmission, dopaminergic	21	3	0.02	15	4.7e-05	9.4e-07
11	GO:0015872	dopamine transport	22	3	0.02	16	5.1e-05	1.1e-06
12	GO:0008637	apoptotic mitochondrial changes	41	3	0.04	20	0.00028	7.4e-06
13	GO:0070997	neuron death	151	4	0.15	21	0.00037	1.0e-05
14	GO:0019538	protein metabolic process	3368	11	3.24	25	0.00055	1.8e-05
15	GO:0051583	dopamine uptake	8	2	0.01	26	0.00067	2.4e-05
16	GO:0006914	autophagy	73	3	0.07	30	0.00107	4.2e-05
17	GO:0070647	protein modification by small protein conjugation or removal	474	5	0.46	32	0.00134	5.7e-05
18	GO:0031399	regulation of protein modification process	815	6	0.78	33	0.00142	6.2e-05
19	GO:0018105	peptidyl-serine phosphorylation	84	3	0.08	34	0.00144	6.5e-05
20	GO:0032225	regulation of synaptic transmission, dopaminergic	14	2	0.01	35	0.00168	7.8e-05
21	GO:0006508	proteolysis	869	6	0.84	36	0.00181	8.9e-05
22	GO:0014059	regulation of dopamine secretion	16	2	0.02	38	0.00194	0.00010
23	GO:0090199	regulation of release of cytochrome c from mitochondria	16	2	0.02	39	0.00194	0.00010
24	GO:0044265	cellular macromolecule catabolic process	556	5	0.54	41	0.00222	0.00012
25	GO:0007628	adult walking behavior	19	2	0.02	42	0.00263	0.00015
26	GO:0010506	regulation of autophagy	20	2	0.02	43	0.00278	0.00016
27	GO:0046928	regulation of neurotransmitter secretion	25	2	0.02	47	0.00410	0.00026
28	GO:0006511	ubiquitin-dependent protein catabolic process	351	4	0.34	49	0.00426	0.00028
29	GO:0042417	dopamine metabolic process	27	2	0.03	50	0.00433	0.00030
30	GO:0016567	protein ubiquitination	388	4	0.37	58	0.00486	0.00040
31	GO:0019220	regulation of phosphate metabolic process	727	5	0.7	64	0.00492	0.00042
32	GO:0031400	negative regulation of protein modification process	165	3	0.16	68	0.00530	0.00048
33	GO:0050806	positive regulation of synaptic transmission	37	2	0.04	71	0.00599	0.00056

*: GO IDs are marked in yellow when only LinkagePD genes are annotated to them but no ParkinTAP candidates.
