# Supplementary material for: Profiling of Parkin-Binding Partners Using Tandem Affinity Purification
Source: PLoS One. 2013 Nov 11;8(11):e78648. doi: 10.1371/journal.pone.0078648 (PMC3823883; doi:10.1371/journal.pone.0078648)
Supplement: Text S1 — Legend Table S3. Selection level pseudocode. (DOCX) [file pone.0078648.s012.docx]

**Supporting Information**

Legend Table S3.

| **Column Label** | **Description** |
| --- | --- |
| Entrez Gene ID | Gene ID from NCBI Entrez |
| HGNC Symbol | Gene Symbol from HGNC |
| Parkin ND | Shortest path network distance to Parkin in SpNet protein-protein interaction network |
| Parkin #SP | Number of shortest paths to Parkin in SpNet protein-protein interaction network |
| MonogenicPD ND | Minimum shortest path network distance to MonogenicPD in SpNet protein-protein interaction network |
| MonogenicPD #ND | Number of shortest paths to MonogenicPD with minimum value in SpNet protein-protein interaction network |
| MonogenicPD #SP | Total number of shortest paths to MonogenicPD with minimum value in SpNet protein-protein interaction network |
| iMonogenicPD | Gene symbols of interacting MonogenicPD |
| NotComplex | No complex interaction with other Parkin TAP candidates |
| Dapple_p-value | p-value from DAPPLE, significance of connectivity within ParkinTAP and MonogenicPD on InWeb network |
| Pink1TAP | Pink1 TAP candidate |
| HNetDegree | Number of protein interactions in iRefIndex |
| HNetBDegree | Number of binary protein interactions in iRefIndex |
| HNetCDegree | Number of complex protein interactions in iRefIndex |
| GOComp; GO:0006986 response to unfolded protein | Annotated to GO:0006986 (response to unfolded protein) or children term, GO enriched in MonogenicPD |
| GOComp; GO:0007005 mitochondrion organization | Annotated to GO:0007005 (mitochondrion organization) or children term, GO enriched in MonogenicPD |
| GOComp; GO:0046907 intracellular transport | Annotated to GO:0046907 (intracellular transport) or children term, GO enriched in MonogenicPD |
| GOComp; GO:0051649 establishment of localization in cell | Annotated to GO:0051649 (establishment of localization in cell) or children term, GO enriched in MonogenicPD |
| GOComp; GO:0043623 cellular protein complex assembly | Annotated to GO:0043623 (cellular protein complex assembly) or children term, GO enriched in MonogenicPD |
| GOComp; GO:0006457 protein folding | Annotated to GO:0006457 (protein folding) or children term, GO enriched in MonogenicPD |
| GOComp | Logical OR of "true" values of six previous GOComp columns |
| FunSimMonogenicPD | Functional similarity >= 0.7 to a MonogenicPD protein |
| GOSlimPD | Annotated to GOSlimPD or children term |
| ParkinGS | Overlap with Parkin fly genetic screen |
| Pink1GS | Overlap with PINK1 fly genetic screen |
| CalmodulinIP | Interaction with calmodulin |
| SelectionLevel | Selection level |

**Selection Level Pseudocode**

SelectionLevel = 8

if GOSlimPD

SelectionLevel = 7

if MonogenicPD ND=1 OR FunSimMonogenicPD

SelectionLevel = 6

if MonogenicPD ND=1 AND (NotComplex OR HNetDegree < 50 OR GOComp)

SelectionLevel = 5

if MonogenicPD ND=1 AND (ParkinGS OR Pink1GS)

SelectionLevel = 4

if (MonogenicPD ND=1 AND Pink1TAP)

OR

(MonogenicPD ND=1 AND GOSlimPD)

OR

(FunSimMonogenicPD AND Pink1TAP)

OR

(FunSimMonogenicPD AND GOSlimPD)

SelectionLevel = 3

if MonogenicPD ND=1 AND FunSimMonogenicPD

SelectionLevel = 2

if MonogenicPD ND=1 AND MonogenicPD #ND > 1

SelectionLevel = 1

if ParkinIP

SelectionLevel = 0
